# Media Supplementation with Mannitol and Biotin Enhances Squalene Production of *Thraustochytrium* ATCC 26185 through Increased Glucose Uptake and Antioxidative Mechanisms

**DOI:** 10.3390/molecules27082449

**Published:** 2022-04-11

**Authors:** M. Kashif Ali, Biswarup Sen, Yaodong He, Mohan Bai, Guangyi Wang

**Affiliations:** 1 Center of Marine Environmental Ecology, School of Environmental Science and Engineering, Tianjin University, Tianjin 300072, China; kashif.web@tju.edu.cn (M.K.A.); bsen@tju.edu.cn (B.S.); yaodong.he@tju.edu.cn (Y.H.); 2 College of Life Science, Zhejiang University, Hangzhou 310058, China; bmh@zju.edu.cn; 3 Key Laboratory of Systems Bioengineering (Ministry of Education), Tianjin University, Tianjin 300072, China; 4 Qingdao Institute Ocean Engineering of Tianjin University, Qingdao 266237, China

**Keywords:** thraustochytrids, squalene, supplementation, mannitol, biotin, oxidative stress

## Abstract

Media supplementation with exogenous chemicals is known to stimulate the accumulation of important lipids produced by microalgae and thraustochytrids. However, the roles of exogenous chemicals in promoting and preserving the terpenoids pool of thraustochytrids have been rarely investigated. Here, we realized the effects of two media supplements—mannitol and biotin—on the biomass and squalene production by a thraustochytrid strain (*Thraustochytrium* sp. ATCC 26185) and elucidated their mechanism of action. A significant change in the biomass was not evident with the exogenous addition of these supplements. However, with mannitol (1 g/L) supplementation, the ATCC 26185 culture achieved the best concentration (642 ± 13.6 mg/L) and yield (72.9 ± 9.6 mg/g) of squalene, which were 1.5-fold that of the control culture (non-supplemented). Similarly, with biotin supplementation (0.15 mg/L), the culture showed 459 ± 2.9 g/L and 55.7 ± 3.2 mg/g of squalene concentration and yield, respectively. The glucose uptake rate at 24 h of fermentation increased markedly with mannitol (0.31 g/Lh^−1^) or biotin (0.26 g/Lh^−1^) supplemented culture compared with non-supplemented culture (0.09 g/Lh^−1^). In addition, the reactive oxygen species (ROS) level of culture supplemented with mannitol remained alleviated during the entire period of fermentation while it alleviated after 24 h with biotin supplementation. The ∆ROS with mannitol was better compared with biotin supplementation. The total antioxidant capacity (T-AOC) of the supplemented culture was more than 50% during the late stage (72–96 h) of fermentation. Our study provides the potential of mannitol and biotin to enhance squalene yield and the first lines of experimental evidence for their protective role against oxidative stress during the culture of thraustochytrids.

## 1. Introduction

Squalene, a natural triterpene (2,6,10,15,19,23–hexamethyltetracosa–2,6,10,14,18,22-hexane; C30H50), is a crucial precursor for the biosynthesis of cholesterol, bile acids, and hormones in plants and animals [1,2]. Due to its anticancer, antioxidant, skin hydrating, and immune-stimulating properties, squalene has increasing applications in nutraceutical, pharmaceutical, and cosmetic industries [3,4], with a tremendous market value that is projected to reach USD 184 million by 2025 [5]. As of now, the shark liver is the primary source of squalene, but this has led to increased poaching and massive damage to the environment [6]. Likewise, the drawbacks of plant-based sources include long harvest time, low-production yield, and feed-food competition [7]. Therefore, sustainable production of squalene from renewable sources has been the biggest challenge for the industrial applications of squalene [4]. Interestingly, several thraustochytrid strains have been shown to have great potential for the high-yield production of squalene because of their advantages of fast growth rate and controllable fermentation [8,9,10,11]. Thraustochytrids are a group of unicellular fungus-like marine microorganisms within the kingdom Stramenopila/Heterokonta [12]. However, low production yield remains one of the most important obstacles that limit the large-scale production of squalene using thraustochytrids [13].

To achieve high-yield production of natural products via microbial fermentation, stress-inducing strategies (e.g., nutrition starvation, salinity, temperature) have been commonly adopted [14]. Under such stress conditions, high levels of reactive oxygen species (ROS) are inevitably accumulated in the cells which usually lead to the decreased production of microbial natural products [15]. Furthermore, most microbial strains tend to accumulate free radicals that specifically attack the cell membrane, which is the prime site for squalene storage [16]. The cell membrane damage can decrease the squalene production yield, most likely through intervening regular cellular functions and overuse of cellular energy. Therefore, the reduction of ROS has been one of the most daunting challenges in the microbial production of natural products [17]. Besides, squalene produced by the cell is metabolized and used up as an important intermediate of sterol biosynthesis [2].

To date, a handful of chemicals have been tested on thraustochytrids/microalgae for their antioxidative properties and potential to improve lipid yield by supplying them exogenously. Some of those chemicals include butylated hydroxyanisole [18,19], sesame oil [20,21], ascorbic acid [21,22], flaxseed oil [23], melatonin [19,21], mannitol [21], and butylhydroxytoluene [19,21]. Studies on media supplementation have proven that certain chemicals enhance lipid yield by minimizing intracellular lipid peroxidation [15]. To our knowledge, the influence of exogenous chemicals on the yield of squalene produced by thraustochytrids has been sporadically investigated [8,24,25,26]. More importantly, the mechanism of action of exogenous chemicals on squalene accumulation remains poorly elucidated. 

In this study, we tested two exogenously added chemicals—mannitol and biotin—for their potential role as media supplements in promoting biomass and squalene production by *Thraustochytrium* ATCC 26185. Both mannitol and biotin are low-cost chemicals. Moreover, biotin is a powerful antioxidant and has been shown to improve the cell growth and increase the production of secondary metabolites [27,28]. Mannitol also provides antioxidant and hydrating properties [21,29], and is known to influence fermentation as a fermentable sugar and source of energy for many microbes [30]. Our study provides the first lines of experimental evidence for the possible mechanisms underlying the effects of mannitol and biotin supplementation on squalene yield. The major goal was to neutralize the intracellular ROS and conserve the squalene pool of the ATCC 26185 strain.

## 2. Results and Discussion

### 2.1. Effects of Mannitol and Biotin on Biomass and Squalene Production

**Figure 1:**
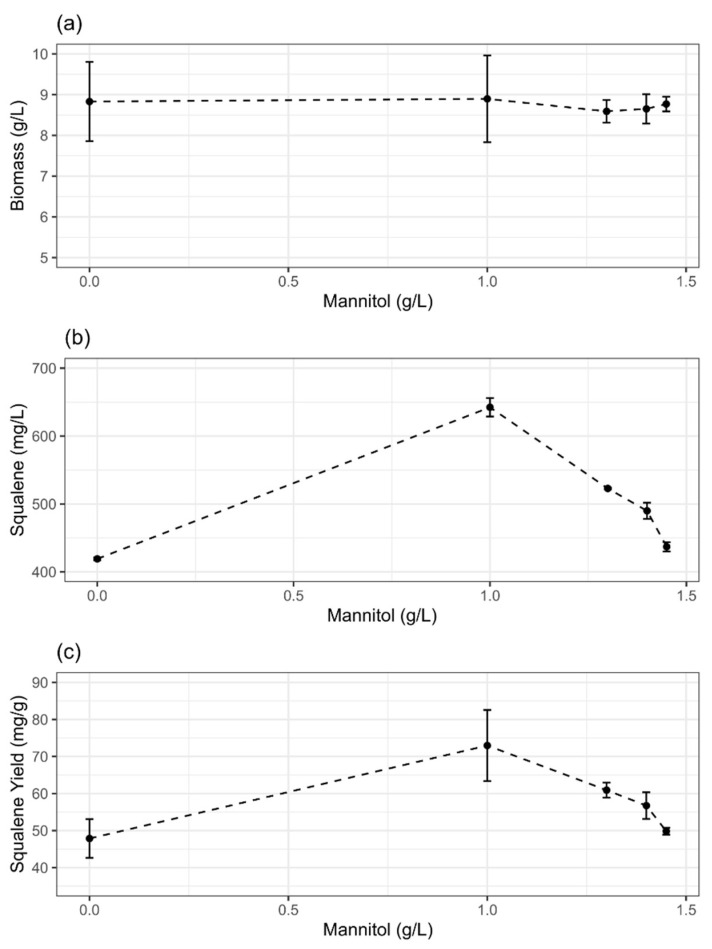
Effects of mannitol supplementation on the (**a**) biomass, (**b**) squalene concentration, and (**c**) squalene yield of ATCC 26185 strain. The data are provided for 72 h grown culture and expressed as mean ± SD of triplicate experiments.

**Figure 2:**
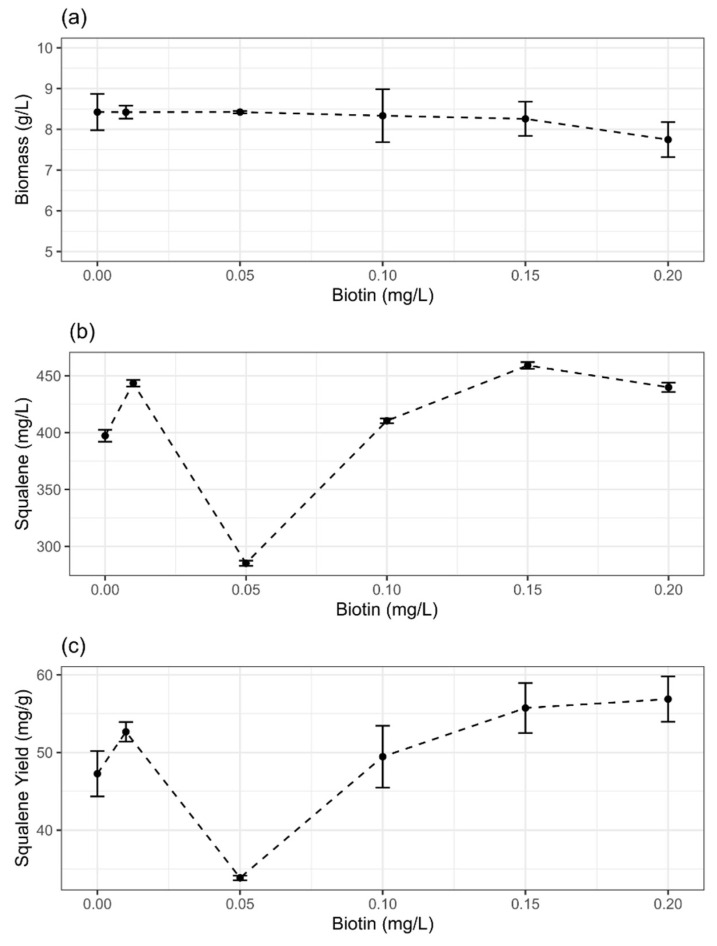
Effects of biotin supplementation on the (**a**) biomass, (**b**) squalene concentration, and (**c**) squalene yield of ATCC 26185 strain. The data are provided for 72 h grown culture and expressed as mean ± SD of triplicate experiments. DMSO was added to the medium without biotin (0 g/L) as a negative control.

The potential of mannitol and biotin supplementation to enhance biomass and squalene production was realized by testing various concentrations of these supplements in batch culture of ATCC 26185 strain. With the increasing concentration of mannitol (Figure 1a) or biotin (Figure 2a), biomass production did not change significantly (*p* > 0.05). The range of biomass was 8.6–8.9 g/L and 7.7–8.4 g/L with mannitol and biotin supplementation, respectively. Similar results were observed when methyl jasmonate was added to the culture of *Schizochytrium mangrovei* in the concentration range of 0 mM to 0.4 mM [24]. While certain supplements (e.g., terbinafine) have been reported to result in the slight inhibition of cell growth of *Schizochytrium* strains [8,25], some other supplements such as ascorbic acid [22] and sesame oil [20] were found to improve microalgal biomass production. Overall, these findings suggest that the response, in terms of cell growth, of thraustochytrid strains to different media supplements can be inconsistent.

The squalene concentrations (Figure 1b and Figure 2b) and yields (Figure 1c and Figure 2c) varied notably along the concentration gradients of mannitol and biotin. Particularly, the responses were dose-dependent in the case of mannitol supplementation. The addition of 1 g/L mannitol or 0.15 mg/L biotin to the initial (t = 0) culture provided the highest concentration and yield of squalene. The concentration and yield of squalene with 1 g/L mannitol supplementation were 642 ± 13.6 mg/L and 72.9 ± 9.6 mg/g, respectively. Similarly, with 0.15 mg/L biotin supplementation, these were 459 ± 2.9 g/L and 55.7 ± 3.2 mg/g, respectively. The supplementation with mannitol provided better improvement (1.5-fold) in squalene production compared with biotin (1.2-fold).

Previous studies have shown that supplements such as methyl jasmonate, butanol, and terbinafine can significantly improve the squalene content of *Schizochytrium* strains. For example, the addition of methyl jasmonate (0.1 mM) to the culture of *S*. *mangrovei* was able to provide a 60% higher squalene yield (1.17 mg/g DCW) than that of the control [24]. Similarly, in the presence of butanol (6 g/L), a 31-fold change (from 0.65 mg/g to 20.09 mg/g) in the squalene content was reported in *S*. *limacinum* B4D1 [26]. With the addition of terbinafine (100 µg/mL) to *S*. *mangrovei* PQ6 culture, an increase of 56.4% in squalene content (96.7 mg/g) compared to the control (61.8 mg/g) was reported [25]. Similarly, at terbinafine concentrations of 100 µg/mL, an increase of 40% in squalene content of *Aurantiochytrium mangrovei* FB3 compared to the control has been reported [8]. Interestingly, our study for the first time showed that a low amount of mannitol (1 g/L) or biotin (0.15 mg/L) can considerably improve the squalene content without affecting the cell growth of ATCC 26186 strain.

### 2.2. Mannitol/Biotin Increases Glucose Uptake Rate

**Figure 3:**
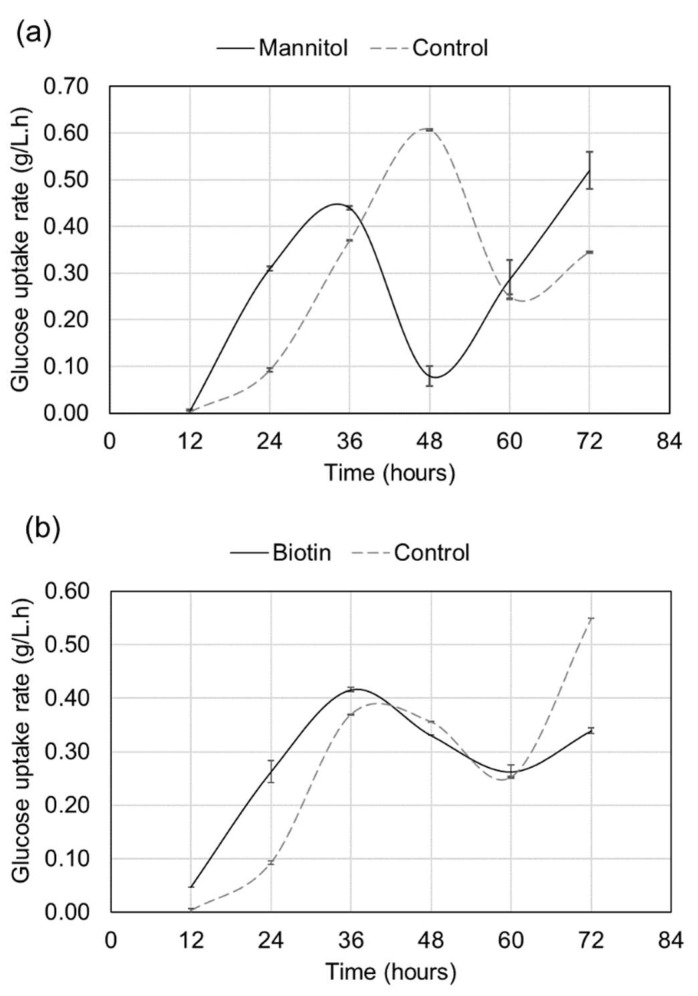
Time course of glucose consumption by ATCC 26185 culture upon (**a**) mannitol (1 g/L) and (**b**) biotin (0.15 mg/L) supplementation at 0 h of fermentation. The data are expressed as the mean ± SD of duplicate experiments.

Glucose uptake rate is considered the first rate-limiting step that drives cellular metabolism, and it depends on the extracellular glucose availability and intracellular metabolic potential [31]. To understand the effect of optimal biotin/mannitol supplementation on the glucose uptake rate, the residual glucose concentration was monitored at regular time intervals during the batch fermentation. The glucose uptake rate fluctuated markedly with fermentation time in both control and supplemented groups, and it peaked twice at different timepoints (Figure 3a,b). The first peak was observed between 36 and 48 h and the second was apparent at 72 h. With mannitol supplementation, the first peak appeared 12 h earlier than that of the control. In addition, the uptake rate at 24 h of fermentation was much greater with mannitol (0.31 g/Lh^−1^) compared with that without mannitol (0.09 g/Lh^−1^). Conversely, the first peak with and without biotin supplementation appeared at the same time point (36 h). Interestingly, consistent with the mannitol supplementation results, the uptake rate at 24 h of fermentation was also much higher with biotin (0.26 g/Lh-1). These results are in agreement with a previous study that reported a significant difference in glucose uptake between non-supplemented and ascorbic acid supplemented cultures after 24 h of fermentation [22]. Taking the findings together, mannitol/biotin supplementation might play a role in increasing the glucose uptake rate of ATCC 26185 culture during the initial phase of fermentation.

Previous studies on a thraustochytrid strain cultured on medium supplemented with flaxseed oil have shown that increased glucose uptake corresponds with higher biomass yields [23]. However, in our study, despite increased glucose uptake rate at 24 h of fermentation, we did not observe a significant increase in biomass yields upon mannitol/biotin supplementation (Figure 1a and Figure 2a); instead, an increased squalene yield was evident (Figure 1c and Figure 2c). Overall, our study suggests that an increased glucose uptake rate induced by mannitol/biotin supplementation might stimulate the metabolic flux towards the squalene biosynthetic pathway resulting in an improved squalene yield. Future research on metabolic flux distribution could provide an understanding of how glucose uptake rate regulates squalene production in thraustochytrid strains. 

### 2.3. Antioxidative Properties of Mannitol and Biotin

**Figure 4:**
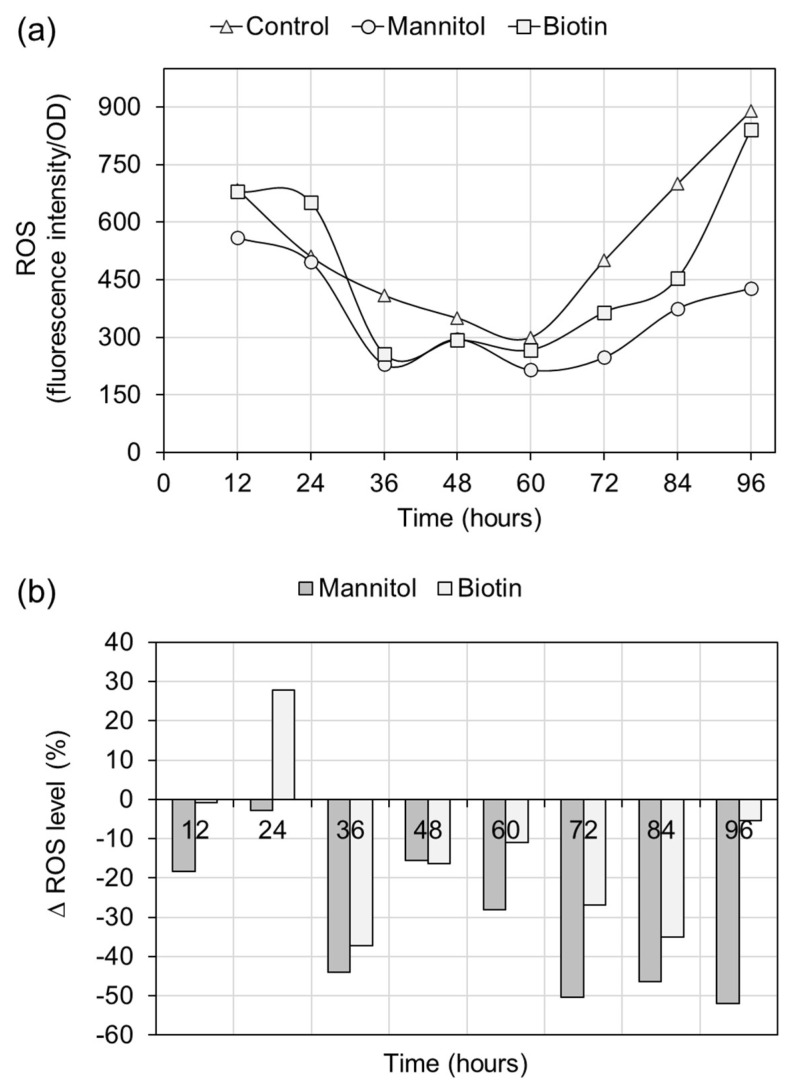
(**a**) Time course profiles of cellular ROS levels in control and supplemented ATCC 26185 cultures, and (**b**) Percentage ∆ROS level of supplemented cultures. The culture was supplemented with 1 g/L mannitol or 0.15 mg/L biotin at 0 h of fermentation.

To ascertain whether mannitol and biotin exhibit antioxidative properties, we compared the cellular levels of ROS and total antioxidant capacity (T-AOC) between control and supplemented cultures of the ATCC 26185 strain. The time courses of specific fluorescence intensity of non-supplemented and supplemented groups somewhat indicated lower ROS levels in the latter groups (Figure 4a). To further understand the extent of the alleviation in ROS levels upon mannitol/biotin supplementation, the differences in the ROS levels between the control and supplemented cultures were evaluated (Figure 4b). The results revealed that the ROS level remained alleviated during the entire period of fermentation when the culture was supplemented with mannitol (1 g/L). Particularly, the ROS alleviation (∆ROS) was high (46.4–51.9%) during the late stage (72–96 h) of fermentation. Contrastingly, the ∆ROS of culture supplemented with biotin started only after 24 h of fermentation and was lower than that with mannitol. Overall, our findings suggest that mannitol might provide better protection to the ATCC 26185 cells against oxidative damage caused by the ROS generated during the process of fermentation.

**Figure 5:**
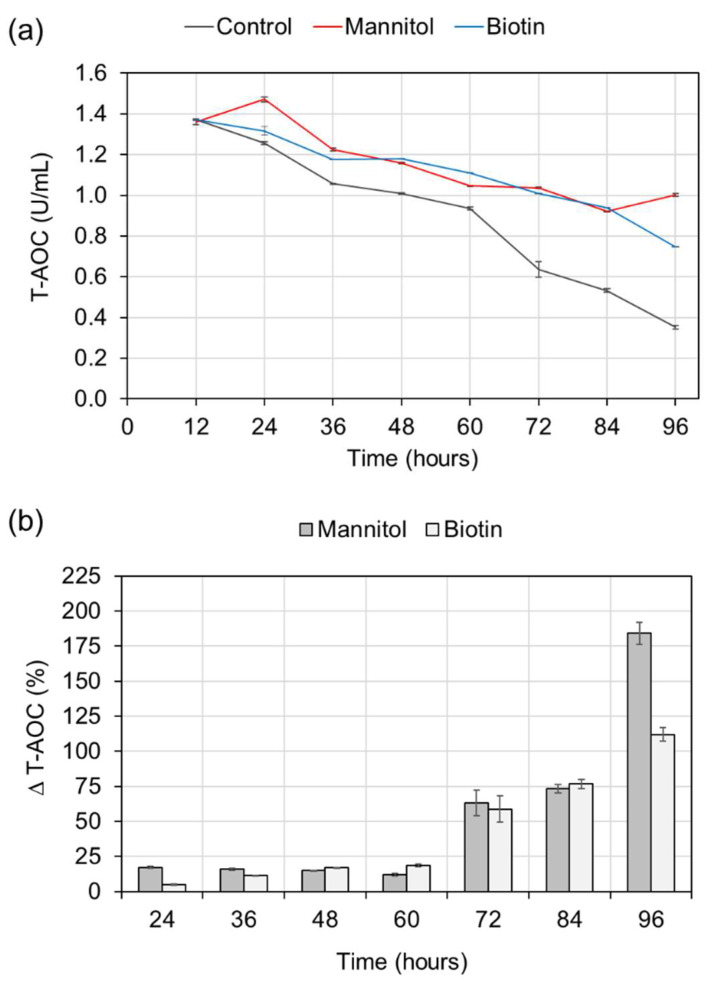
(**a**) Time course profiles of T-AOC in control and supplemented ATCC 26185 cultures, and (**b**) Percentage ∆T-AOC level of supplemented cultures. The culture was supplemented with 1 g/L mannitol or 0.15 mg/L biotin at 0 h of fermentation. The data are expressed as mean ± SD of triplicate measurements.

The T-AOC levels of control and supplemented cultures were found to decline with fermentation time. Interestingly, the supplementation with mannitol or biotin aided the slowdown of the declining trend. Furthermore, the time-course profiles of T-AOC revealed moderately increased levels in the supplemented cultures compared with the control culture (Figure 5a). Particularly, the T-AOC elevation (∆T-AOC) in the supplemented cultures was more than 50% during the late stage (72–96 h) of fermentation. In addition, there was a markedly higher elevation of T-AOC at 96 h with mannitol (∆T-AOC = 184%) than with biotin (∆T-AOC = 112%) supplementation.

Fundamentally, two different types of antioxidative processes are known to function in response to the cellular oxidative stress for protecting against oxidative damage to the cell [32,33]. These include the direct antioxidant scavenging activity, where antioxidants absorb ROS directly and promptly, and the expression of genes for antioxidant enzymes such as superoxide dismutase (SOD). The latter is a relatively indirect way to reduce cellular oxidative stress. Previous studies have shown that mannitol treatment significantly enhances the SOD activity by activation of already synthesized enzyme isoforms such as Mn-SOD3, Cu/Zn-SOD3, and Cu/ZnSOD4 and by inducing the synthesis of new isoforms such as Mn-SOD2 and Cu/Zn-SOD1 [34]. Our results provide evidence for the contribution of mannitol and biotin in the enhancement of both the antioxidative processes (i.e., ROS alleviation and T-AOC elevation) within the cell.

## 3. Materials and Methods

### 3.1. Strain and Culture Conditions

*Thraustochytrium* sp. ATCC 26185 was purchased from American Type Culture Collection (ATCC) (Manassas, VA, USA). The strain was maintained on agar plates containing SQU medium: 30 g glucose, 2 g yeast extract, 2 g monosodium glutamate (MSG), 0.2 g (NH_4_)_2_SO_4_, 0.3 g KH_2_PO_4_, 25 g NaCl, 1 g KCl, 5 g MgSO_4_·7H_2_O, 0.1 g NaHCO_3_, 0.3 g CaCl_2_, 2.9 mg FeCl_3_·6H_2_O, 0.02 mg CuSO_4_·5H_2_O, 0.26 mg CoCl·6H_2_O, 0.6 mg ZnSO_4_·7H_2_O, 8.6 mg MnSO_4_·H_2_O, and 20 g agar in one liter of distilled water [24]. The inoculated agar plate was kept at 28 °C and sub-cultured every four weeks. The initial seed culture was prepared by transferring a single colony from the agar plate into an Erlenmeyer flask containing fresh SQU medium and then incubating the flask for 48 h at 28 °C on an orbital shaker set at 170 rpm.

### 3.2. Batch Experiments

Various concentrations of mannitol (g/L: 0, 1.0, 1.3, 1.4, and 1.45) and biotin (mg/L: 0.01, 0.05, 0.1, 0.15, and 0.2) were tested for their effects on the biomass and squalene production under batch conditions. The concentration ranges of mannitol and biotin were based on previous studies [29,35,36]. The stock solutions of mannitol (5 g/100 mL) and biotin (100 mg/L) were prepared in water and DMSO, respectively, and stored in the dark at −20 °C. The seed culture was transferred to 100 mL shake flask containing 50 mL of SQUA medium: 25 g/L glucose, 5 g/L NaCl, 5 g/L MgSO_4_.7H_2_O, 2 g/L MSG, 2.5 g/L yeast extract, 1 g/L KCl, 0.3 g/L CaCl_2_, 0.3 g/L KH_2_PO_4_, 0.1 g/L NaHCO_3_, and micronutrients (see Section 3.1). An appropriate volume of mannitol/biotin was added to the culture medium to achieve its desired final concentration. The addition of mannitol/biotin at 0 h of fermentation provided the optimum surge (data not shown). All experiments were carried out at 28 °C in an orbital shaker set at 170 rpm.

### 3.3. Quantification of Biomass, Squalene, and Residual Glucose

The dry cell weight (DCW) and squalene content were measured following the procedures described in our previous study [13]. To determine the concentration of residual glucose, one ml of culture broth was collected every 12 h until 72 h and centrifuged for 10 min at 10,000 rpm and 4 °C. The resulting supernatant was diluted (10×) with distilled water, and its glucose content was measured using the Glu KIT (Biosino Bio-Technology and Science Inc., Beijing, China) as per the manufacturer’s instructions. The color intensity of the reaction mixture was measured at 505 nm in a spectrophotometer (Multiskan GO, Thermo Scientific, Waltham, MA, USA) and the concentration (g/L) was determined from the glucose standard curve. The glucose uptake rate was calculated by dividing the difference of residual glucose concentrations with the time interval between two consecutive time points.

### 3.4. Determination of Intracellular ROS and T-AOC Levels

The intracellular ROS and T-AOC levels were measured following the procedures described in our previous study [17]. The ∆ROS level and ∆T-AOC were calculated as a percentage of the difference between the control and supplemented groups.

### 3.5. Statistical Analysis

The group means of biomass and squalene concentration and yield were statistically analyzed at the alpha level of 0.05 using ANOVA. All statistical tests and visualization were done in R version 4.0.4 [37]. 

## 4. Conclusions

This study provides a practical strategy in which exogenous addition of mannitol or biotin to culture medium enhances the squalene content of *Thraustochytrium* ATCC 26185. Although these supplements did not notably improve the biomass yield, they could considerably enhance the squalene yield. The uptake rate of the medium carbon source (glucose) for the supplemented culture was significantly higher in the initial stage of fermentation. Furthermore, these supplements exhibited potential antioxidative properties; particularly, mannitol showed better ROS quenching ability than biotin. These findings suggest that possibly through the mechanism of increased glucose uptake and added protection against oxidative stress, mannitol and biotin might contribute to the enhanced squalene content of the ATCC 26185 strain.

## Data Availability

Not applicable.

## Sample Availability

Not available.

## References

[1] Smith T.J. (2000). Squalene: Potential chemopreventive agent. Expert Opin. Investig. Drugs.

[2] Ghimire G.P., Thuan N.H., Koirala N., Sohng J.K. (2016). Advances in biochemistry and microbial production of squalene and its derivatives. J. Microbiol. Biotechnol..

[3] Reddy L.H., Couvreur P. (2009). Squalene: A natural triterpene for use in disease management and therapy. Adv. Drug Del. Rev..

[4] Gohil N., Bhattacharjee G., Khambhati K., Braddick D., Singh V. (2019). Engineering Strategies in Microorganisms for the Enhanced Production of Squalene: Advances, Challenges and Opportunities. Front. Bioeng. Biotechnol..

[5] MARKETSANDMARKETS Squalene Market by Source Type (Animal Source (Shark Liver Oil), Vegetable Source (Olive Oil, Palm Oil, Amaranth Oil), Biosynthetic (GM Yeast]), End-use Industry (Cosmetics, Food, and Pharmaceuticals), and Region-Global Forecast to 2025. https://www.marketsandmarkets.com/Market-Reports/squalene-market-542345.html.

[6] Xu W., Ma X., Wang Y. (2016). Production of squalene by microbes: An update. World J. Microbiol. Biotechnol..

[7] Lozano-Grande M.A., Gorinstein S., Espitia-Rangel E., Dávila-Ortiz G., Martínez-Ayala A.L. (2018). Plant Sources, Extraction Methods, and Uses of Squalene. Int. J. Agron..

[8] Fan K.W., Aki T., Chen F., Jiang Y. (2010). Enhanced production of squalene in the thraustochytrid *Aurantiochytrium mangrovei* by medium optimization and treatment with terbinafine. World J. Microbiol. Biotechnol..

[9] Chen G., Fan K.W., Lu F.P., Li Q., Aki T., Chen F., Jiang Y. (2010). Optimization of nitrogen source for enhanced production of squalene from thraustochytrid *Aurantiochytrium* sp.. New Biotechnol..

[10] Nakazawa A., Matsuura H., Kose R., Kato S., Honda D., Inouye I., Kaya K., Watanabe M.M. (2012). Optimization of culture conditions of the thraustochytrid *Aurantiochytrium* sp. strain 18W-13a for squalene production. Bioresour. Technol..

[11] Otagiri M., Khalid A., Moriya S., Osada H., Takahashi S. (2017). Novel squalene-producing thraustochytrids found in mangrove water. Biosci. Biotechnol. Biochem..

[12] Leyland B., Leu S., Boussiba S. (2017). Are Thraustochytrids algae?. Fungal Biol..

[13] Zhang A., Xie Y., He Y., Wang W., Sen B., Wang G. (2019). Bio-based squalene production by *Aurantiochytrium* sp. through optimization of culture conditions, and elucidation of the putative biosynthetic pathway genes. Bioresour. Technol..

[14] Singh P., Kumari S., Guldhe A., Misra R., Rawat I., Bux F. (2016). Trends and novel strategies for enhancing lipid accumulation and quality in microalgae. Renew. Sustain. Energy Rev..

[15] Zhang S., He Y., Sen B., Wang G. (2020). Reactive oxygen species and their applications toward enhanced lipid accumulation in oleaginous microorganisms. Bioresour. Technol..

[16] Meng Y., Shao X., Wang Y., Li Y., Zheng X., Wei G., Kim S.-W., Wang C. (2020). Extension of cell membrane boosting squalene production in the engineered Escherichia coli. Biotechnol. Bioeng..

[17] Zhang S., He Y., Sen B., Chen X., Xie Y., Keasling J.D., Wang G. (2018). Alleviation of reactive oxygen species enhances PUFA accumulation in *Schizochytrium* sp. through regulating genes involved in lipid metabolism. Metab. Eng. Commun..

[18] Sui X., Niu X., Shi M., Pei G., Li J., Chen L., Wang J., Zhang W. (2014). Metabolomic analysis reveals mechanism of antioxidant butylated hydroxyanisole on lipid accumulation in *Crypthecodinium cohnii*. J. Agric. Food. Chem..

[19] Cui N., Xiao J., Feng Y., Zhao Y., Yu X., Xu J.-W., Li T., Zhao P. (2021). Antioxidants enhance lipid productivity in Heveochlorella sp. Yu. Algal Res..

[20] Liu B., Liu J., Sun P., Ma X., Jiang Y., Chen F. (2015). Sesamol enhances cell growth and the biosynthesis and accumulation of docosahexaenoic acid in the microalga *Crypthecodinium cohnii*. J. Agric. Food. Chem..

[21] Zhang S., Chen X., Sen B., Bai M., He Y., Wang G. (2021). Exogenous Antioxidants Improve the Accumulation of Saturated and Polyunsaturated Fatty Acids in Schizochytrium sp. PKU#Mn4. Mar. Drugs.

[22] Ren L., Sun X., Ji X., Chen S., Guo D., Huang H. (2017). Enhancement of Docosahexaenoic Acid Synthesis by Manipulation of Antioxidant Capacity and Prevention of Oxidative Damage in *Schizochytrium* sp.. Bioresour. Technol..

[23] Gaffney M., O’Rourke R., Murphy R. (2014). Manipulation of fatty acid and antioxidant profiles of the microalgae *Schizochytrium* sp. through flaxseed oil supplementation. Algal Res..

[24] Yue C.-J., Jiang Y. (2009). Impact of methyl jasmonate on squalene biosynthesis in microalga *Schizochytrium mangrovei*. Process Biochem..

[25] Lan Anh H., Ha N.C., Thom L.T., Hong D. (2016). Optimization of culture conditions and squalene enrichment from heterotrophic marine microalga *Schizochytrium mangrovei* PQ6 for squalene production. Res. J. Biotechnol..

[26] Zhang K., Chen L., Liu J., Gao F., He R., Chen W., Guo W., Chen S., Li D. (2017). Effects of butanol on high value product production in *Schizochytrium limacinum* B4D1. Enzym. Microb. Technol..

[27] Lal D.N., Srivastava A.S. (1982). Effect of vitamins on microbial production of citric acid by *Aspergillus niger*. Zentralbl. Mikrobiol..

[28] Ren L.J., Wei P., Feng Y., Ji X.J., Huang H. (2012). Effect of biotin and cerulenin addition on DHA production by *Schizochytrium* sp.. Chin. J. Bioproc. Eng..

[29] André P., Villain F. (2017). Free radical scavenging properties of mannitol and its role as a constituent of hyaluronic acid fillers: A literature review. Int. J. Cosmet. Sci..

[30] Zhang M., Gu L., Cheng C., Ma J., Xin F., Liu J., Wu H., Jiang M. (2018). Recent advances in microbial production of mannitol: Utilization of low-cost substrates, strain development and regulation strategies. World J. Microbiol. Biotechnol..

[31] Kim D., Song J.-Y., Hahn J.-S. (2015). Improvement of glucose uptake rate and production of target chemicals by overexpressing hexose transporters and transcriptional activator Gcr1 in *Saccharomyces cerevisiae*. Appl. Environ. Microbiol..

[32] Dinkova-Kostova A.T., Talalay P. (2008). Direct and indirect antioxidant properties of inducers of cytoprotective proteins. Mol. Nutr. Food Res..

[33] Halliwell B. (2007). Biochemistry of oxidative stress. Biochem. Soc. Trans..

[34] Seckin B., Sekmen A.H., Türkan İ. (2008). An Enhancing Effect of Exogenous Mannitol on the Antioxidant Enzyme Activities in Roots of Wheat Under Salt Stress. J. Plant Growth Regul..

[35] Li Q., Chen J., Liu G.H., Xu X., Zhang Q., Wang Y., Yuan J., Li Y., Qi L., Wang H. (2021). Effects of biotin on promoting anammox bacterial activity. Sci. Rep..

[36] Jameel M.A.-K. (2001). Optimization of Biotin and Thiamine Requirements for Somatic Embryogenesis of Date Palm (*Phoenix dactylifera* L.). Vitr. Cell. Dev. Biol. Plant.

[37] R Development Core Team (2020). R: A Language and Environment for Statistical Computing.

